# Supplementary material for: Conservation agriculture improves soil health and sustains crop yields after long-term warming
Source: Nat Commun. 2024 Oct 10;15:8785. doi: 10.1038/s41467-024-53169-6 (PMC11467207; doi:10.1038/s41467-024-53169-6)
Supplement: Supplementary file 1 — Supplementary Information [file 41467_2024_53169_MOESM1_ESM.pdf]

Supplementary information for

Conservation agriculture improves soil health and sustains crop yields after long-term warming

Jialing Teng, Ruixing Hou, Jennifer A. J. Dungait, Guiyao Zhou, Yakov Kuzyakov, Jingbo Zhang, Jing Tian, Zhenling Cui, Fusuo Zhang, Manuel Delgado-Baquerizo

This file includes,

Supplementary Figures 1-9

Supplementary Tables 1-3

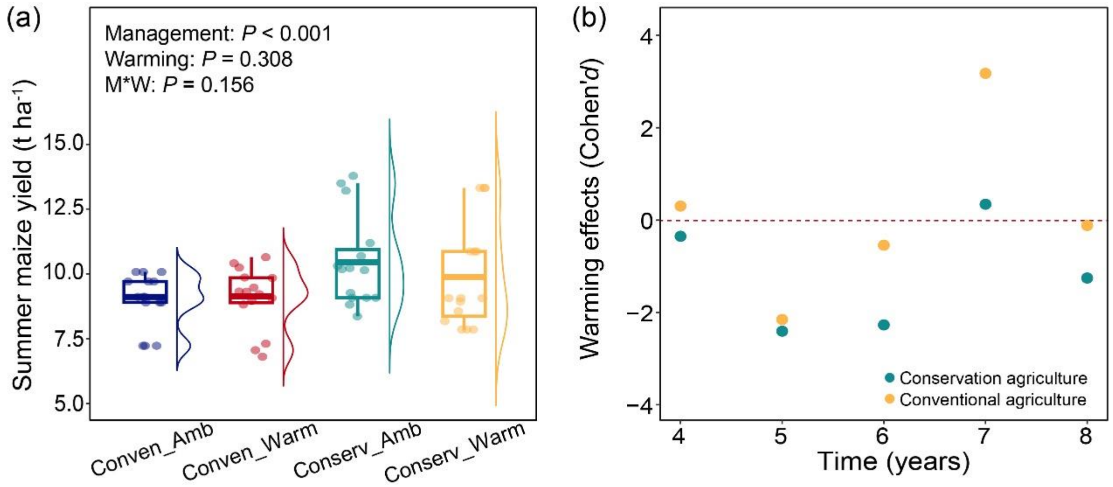

Supplementary Figure 1 Effects of warming and management on summer maize yield. a Average summer maize yield. Boxplots display the mean (horizontal line), the 25th and 75th percentiles (colored box), the minimum and maximum (whiskers). The data were analyzed based on five sampling years (n = 15 individual sample per treatment). Statistical analysis was performed using linear mixed model with sampling time as random factors. All reported *P* values result from two-sided statistical tests. Asterisk indicate significant differences in the warming effect of the individual management system as compared with their matched ambient condition. **b** Shift in the effect size of warming on crop yield over time for conservation and conventional agriculture, respectively. Linear regression model with two-sided test was used for the statistical analysis, and adjusted R-squared was used. Relationships are denoted with solid lines and fit statistics (R^2 and *P* values) for each management practice. The solid line represents the significant linear regression ($P < 0.05$), and the gray shading indicates the 95% confidence intervals. All reported *P* values result from two-sided statistical tests with *$P < 0.05$, ** $P < 0.01$, and *** $P < 0.001$. Conserv-Amb, conservation agriculture without warming; Conserv-Warm, conservation agriculture with warming; Conven-Amb, conventional agriculture without warming; Conven-Warm, conventional agriculture with warming.

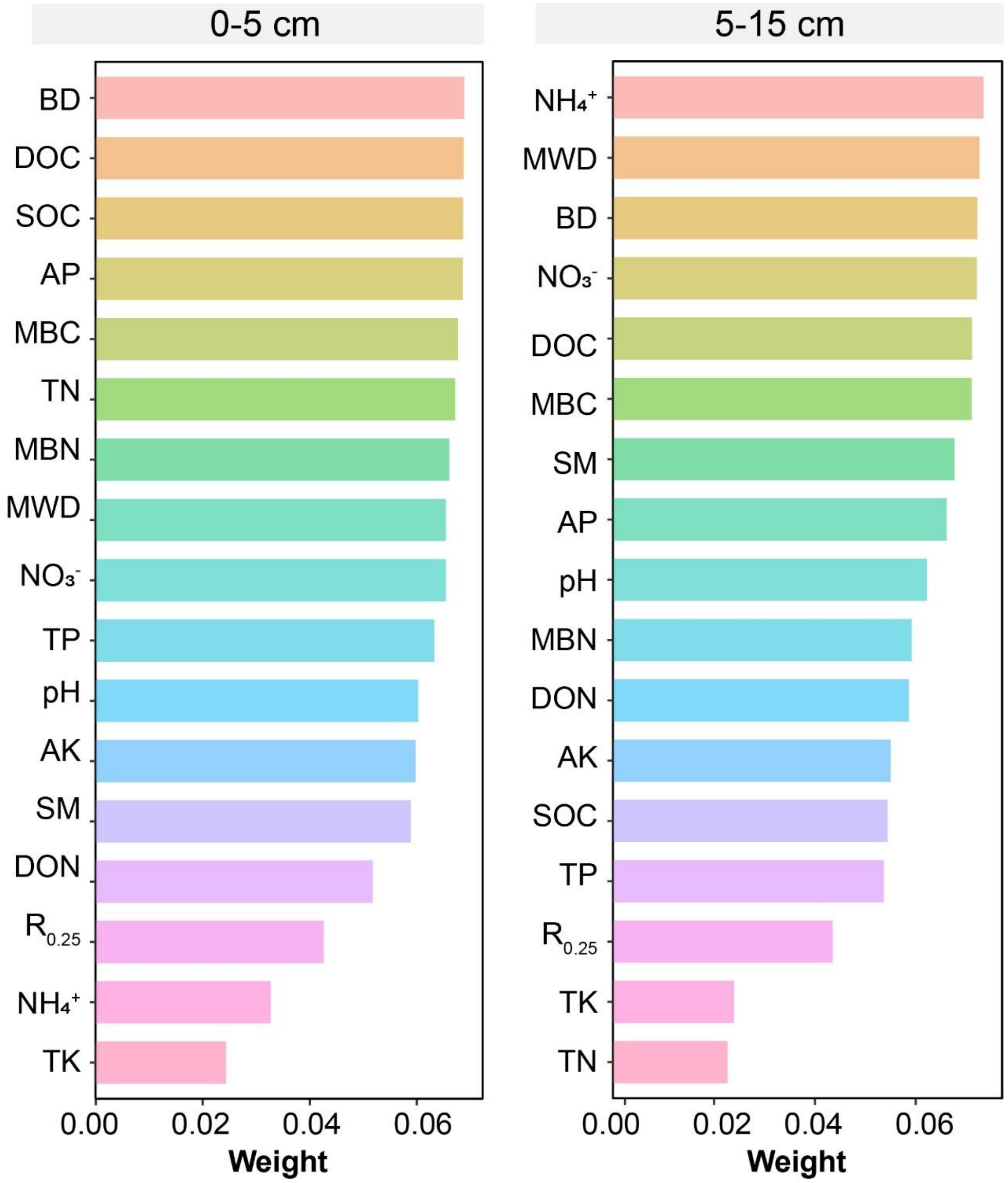

Supplementary Figure 2 Weighting factors based on Principal Component Analysis (PCA) of eigenvectors for the different soil indicators to calculate the soil health score. MWD, mean weight diameter; $R_{0.25}$, aggregate content with particle size larger than 0.25 mm; SM, soil moisture; BD, bulk density; DOC, dissolved organic carbon; SOC, soil organic carbon; NH_4^+-N, ammonium-nitrogen; NO_3^-N, nitrate-nitrogen; DON, dissolved organic nitrogen; TN, total nitrogen; AP, available phosphorus; TP, total phosphorus; AK, available potassium; TK, total potassium; MBC, microbial biomass carbon; MBN, microbial biomass nitrogen.

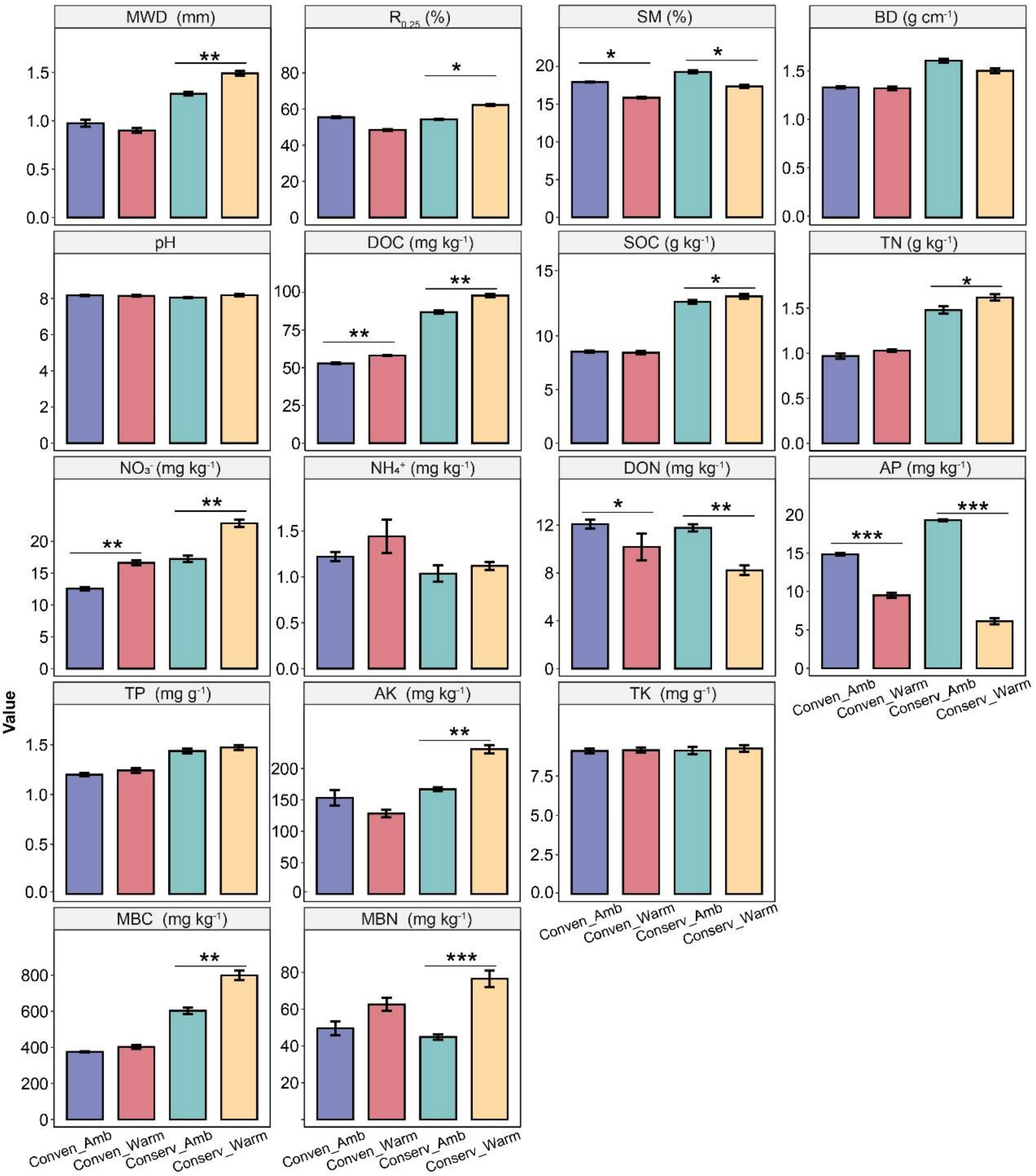

Supplementary Figure 3 Effects of warming and management on individual soil attribute of 0-5 cm soil depth. All parameters were analyzed based on soil sampling in 2020 (n = 4 per treatment). Statistical analysis was performed using two-way ANOVA analysis. Reported *P* values result from two-sided statistical tests with *$P < 0.05$, ** $P < 0.01$, and *** $P < 0.001$. Conserv-Amb, conservation agriculture without warming; Conserv-Warm, conservation agriculture with warming; Conven-Amb, conventional agriculture without warming; Conven-Warm, conventional agriculture with warming. MWD, mean weight diameter; $R_{0.25}$, aggregate content with particle size larger than 0.25 mm; SM, soil moisture; BD, bulk density; DOC, dissolved organic carbon; SOC, soil organic carbon; NH_4^+-N, ammonium-nitrogen; NO_3^-N, nitrate-nitrogen; DON, dissolved organic nitrogen; TN, total nitrogen; AP, available phosphorus; TP, total phosphorus; AK, available potassium; TK, total potassium; MBC, microbial biomass carbon; MBN, microbial biomass nitrogen.

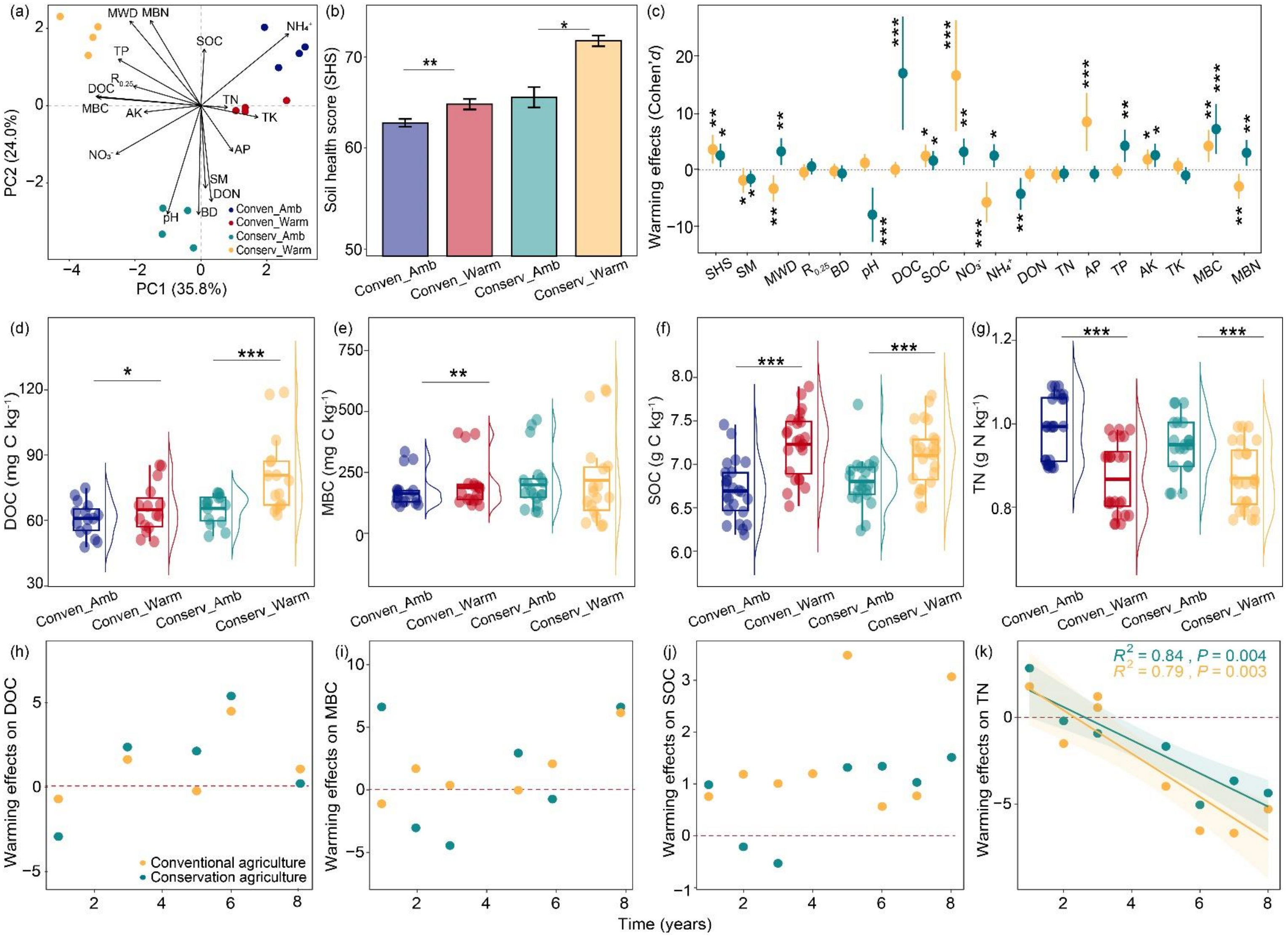

(a)
MWD MBN
SOC
NH4+
TP
R0.25
DOC
MBC
AK
TN
TK
AP
NO3-
SM
DON
BD
pH
Conven_Amb
Conven_Warm
Conserv_Amb
Conserv_Warm
PC1 (35.8%)
PC2 (24.0%)
(b)
Soil health score (SHS)
Conven_Amb
Conven_Warm
Conserv_Amb
Conserv_Warm
(c)
Warming effects (Cohen'd)
SHS SM MWD R0.25 BD pH DOC SOC NO3- NH4+ DON TN AP TP AK TK MBC MBN
(d)
DOC (mg C kg-1)
(e)
MBC (mg C kg-1)
(f)
SOC (g C kg-1)
(g)
TN (g N kg-1)
Conven_Amb
Conven_Warm
Conserv_Amb
Conserv_Warm
(h)
Warming effects on DOC
Conventional agriculture
Conservation agriculture
(i)
Warming effects on MBC
(j)
Warming effects on SOC
(k)
Warming effects on TN
R2 = 0.84 , P = 0.004
R2 = 0.79 , P = 0.003
Time (years)

Supplementary Figure 4 Effects of warming and management on soil attributes and health score of 5-15 cm soil depth. a Two dimensions of Principal component analysis (PCA) for eigenvalues of the seventeen soil attributes, including MWD, $R_{0.25}$, SM, BD, pH, DOC, SOC, NH_4^+-N, NO_3^--N, DON, TN, AP, TP, AK, TK, MBC, and MBN. All parameters were analyzed based on soil sampling in 2020 (n = 4 per treatment). **b** Effects of warming and management on soil health score. Data are presented with mean values ± s.e.m. Statistical analysis was performed using two-way ANOVA analysis. Soil health was evaluated based on soil sampling in 2020 (n = 4 per treatment). **c** Estimates (±95% CI) of the warming effects on soil attributes depending on management. Error bars indicated the 95% confidence interval (CI). If the CI did not overlap with zero, a response was considered to be significant. **d-g** Average of key soil attributes over 8 years determined by linear mixed model. **h-k** Temporal changes of warming effects on key soil properties over time depending on management systems. The effect size was estimated by Cohen' *d*. Significant trends ($P < 0.05$) are shown with solid regression lines. All reported *P* values result from two-sided statistical tests with *$P < 0.05$, ** $P < 0.01$, and *** $P < 0.001$. Conserv-Amb, conservation agriculture without warming; Conserv-Warm, conservation agriculture with warming; Conven-Amb, conventional agriculture without warming; Conven-Warm, conventional agriculture with warming. MWD, mean weight diameter; $R_{0.25}$, aggregate content with particle size larger than 0.25 mm; SM, soil moisture; BD, bulk density; DOC, dissolved organic carbon; SOC, soil organic carbon; NH_4^+-N, ammonium-nitrogen; NO_3^-N, nitrate-nitrogen; DON, dissolved organic nitrogen; TN, total nitrogen; AP, available phosphorus; TP, total phosphorus; AK, available potassium; TK, total potassium; MBC, microbial biomass carbon; MBN, microbial biomass nitrogen.

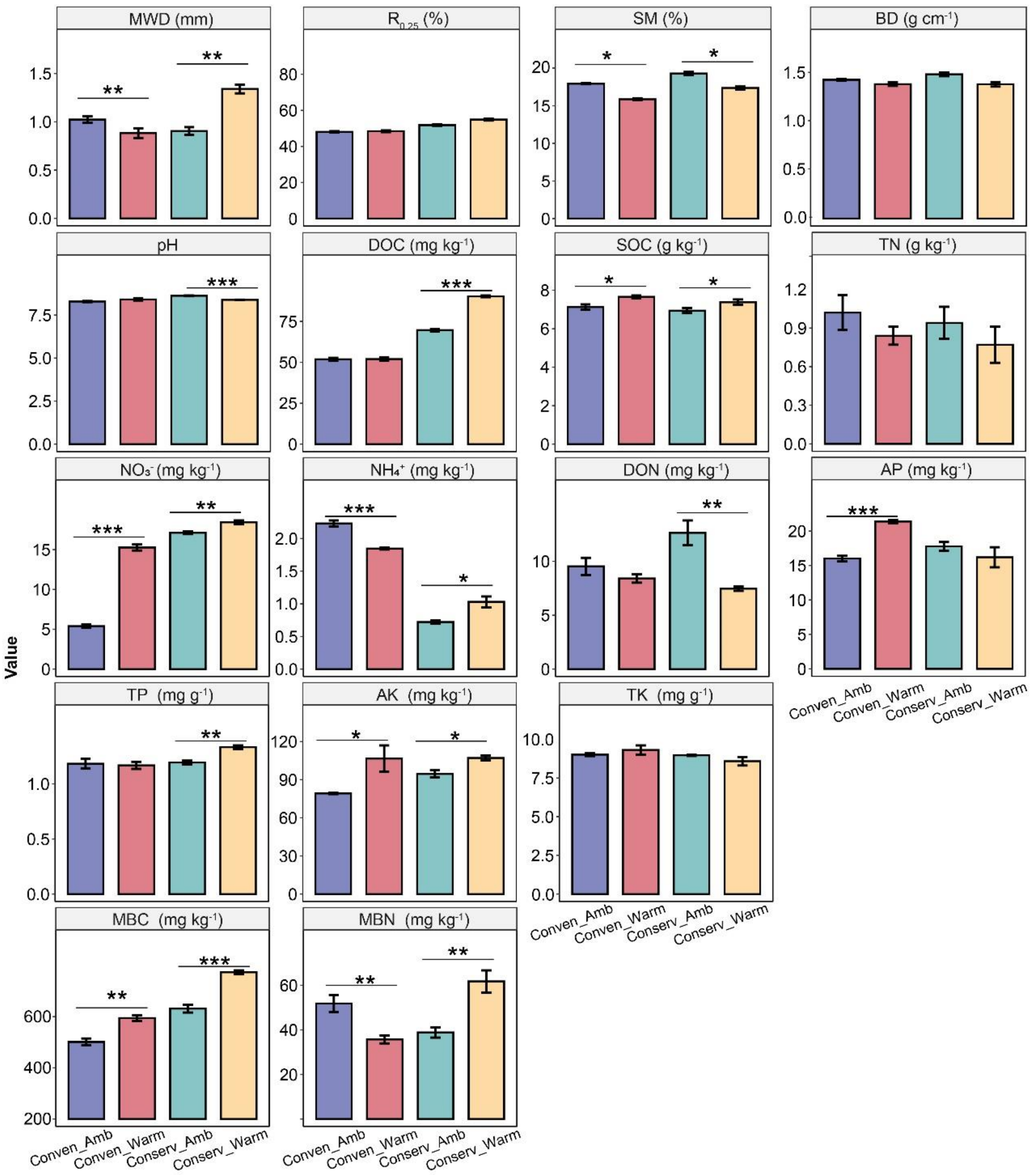

Supplementary Figure 5 Effects of warming and management on individual soil attribute of 5-15 cm soil depth. All parameters were analyzed based on soil sampling in 2020 (n = 4 per treatment). Reported *P* values result from two-sided statistical tests with **P* < 0.05, ** *P* < 0.01, and *** *P* < 0.001. Conserv-Amb, conservation agriculture without warming; Conserv-Warm, conservation agriculture with warming; Conven-Amb, conventional agriculture without warming; Conven-Warm, conventional agriculture with warming. MWD, mean weight diameter; $R_{0.25}$, aggregate content with particle size larger than 0.25 mm; SM, soil moisture; BD, bulk density; DOC, dissolved organic carbon; SOC, soil organic carbon; NH_4^+-N, ammonium-nitrogen; NO_3^-N, nitrate-nitrogen; DON, dissolved organic nitrogen; TN, total nitrogen; AP, available phosphorus; TP, total phosphorus; AK, available potassium; TK, total potassium; MBC, microbial biomass carbon; MBN, microbial biomass nitrogen.

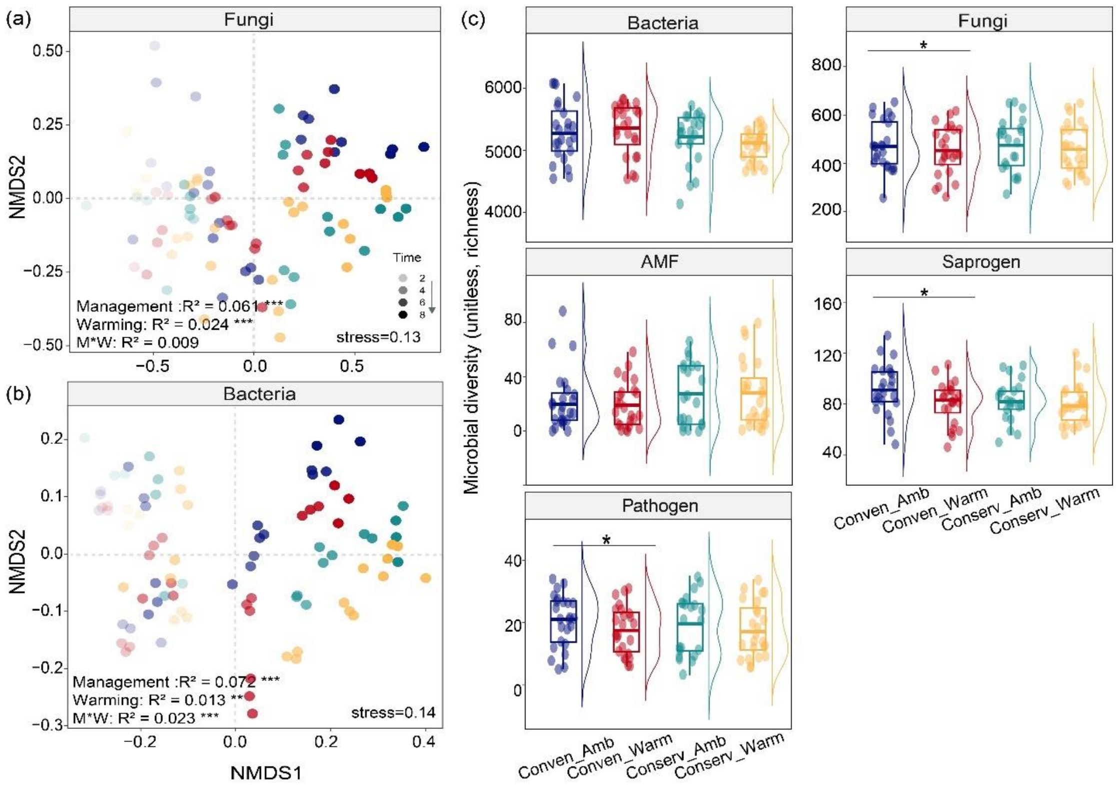

Supplementary Figure 6 Effects of warming and management on microbial diversity and community composition at 5-15 cm soil depth. a, b Nonmetric multidimensional scaling (NMDS) ordination of soil fungal and bacterial communities based on the Bray-Curtis dissimilarity. Statistical analysis was performed using nested permutational multivariate analysis of variance (nested PERMANOVA) analysis. **c** Effects of warming and management on microbial richness of soil total fungi, fungal guild, and bacteria. Boxplots display the mean (horizontal line), the 25th and 75th percentiles (colored box), the minimum and maximum (whiskers). Data were analyzed based on eight sampling years (n = 24 per treatment). Statistical analysis was performed using linear mixed model with sampling time as random factors. All reported *P* values result from two-sided statistical tests with **P* < 0.05, ** *P* < 0.01, and *** *P* < 0.001. Conserv-Amb, conservation agriculture without warming; Conserv-Warm, conservation agriculture with warming; Conven-Amb, conventional agriculture without warming; Conven-Warm, conventional agriculture with warming.

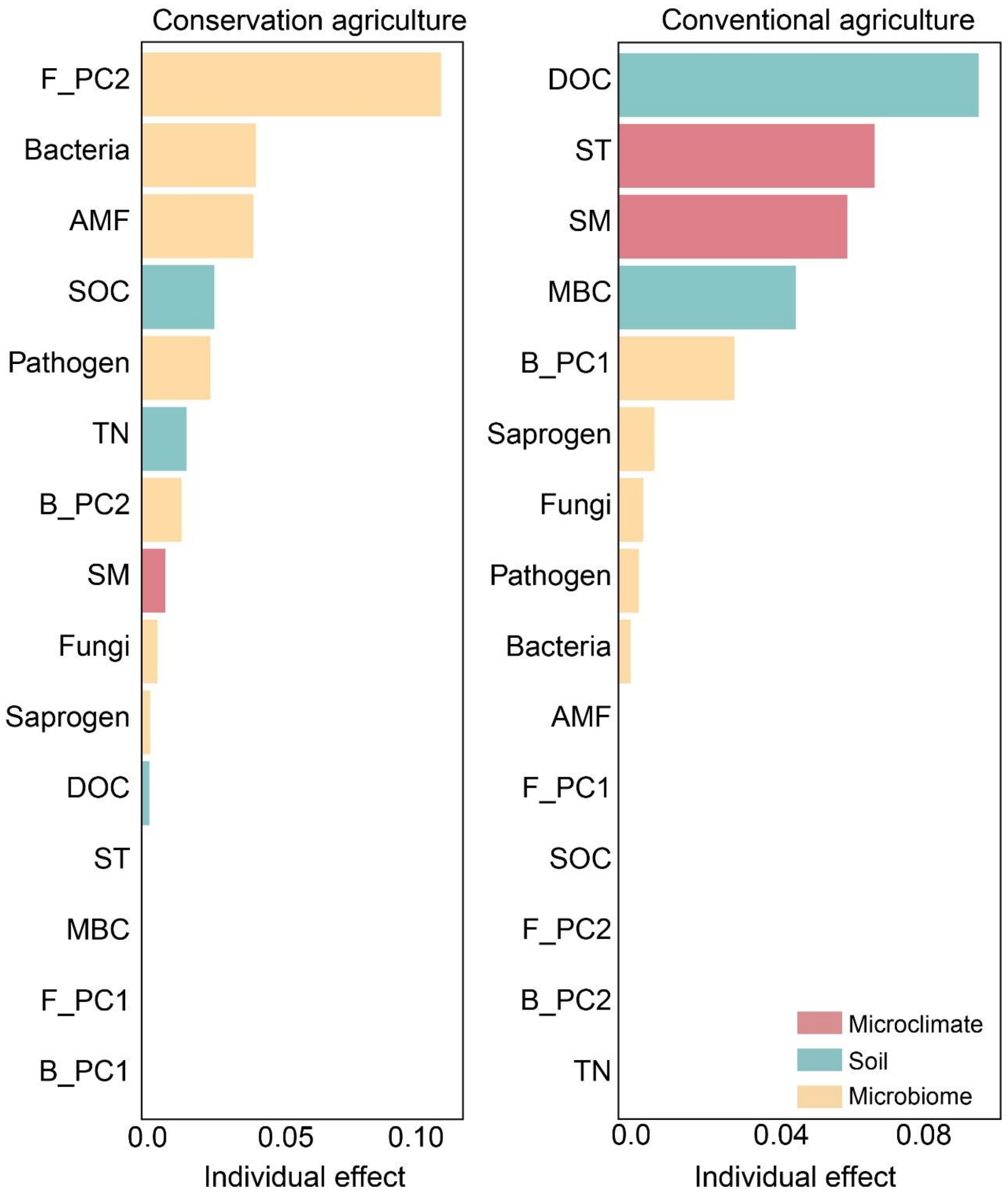

Supplementary Figure 7 Effects of abiotic and biotic factors on winter wheat yield of 5-15 cm soil depth. Statistical analysis was performed using linear mixed model with sampling time as random factors. All reported *P* values result from two-sided statistical tests with **P* < 0.05, ** *P* < 0.01, and *** *P* < 0.001. ST, soil temperature; SM, soil moisture; DOC, dissolved organic carbon; MBC, microbial biomass carbon; SOC, soil organic carbon; TN, total nitrogen. Bacteria, Fungi, AMF, Saprogen, and Pathogen indicated richness of microbial group. F_PC1, F_PC2, B_PC1, and B_PC2 indicated fungal and bacterial community composition, respectively.

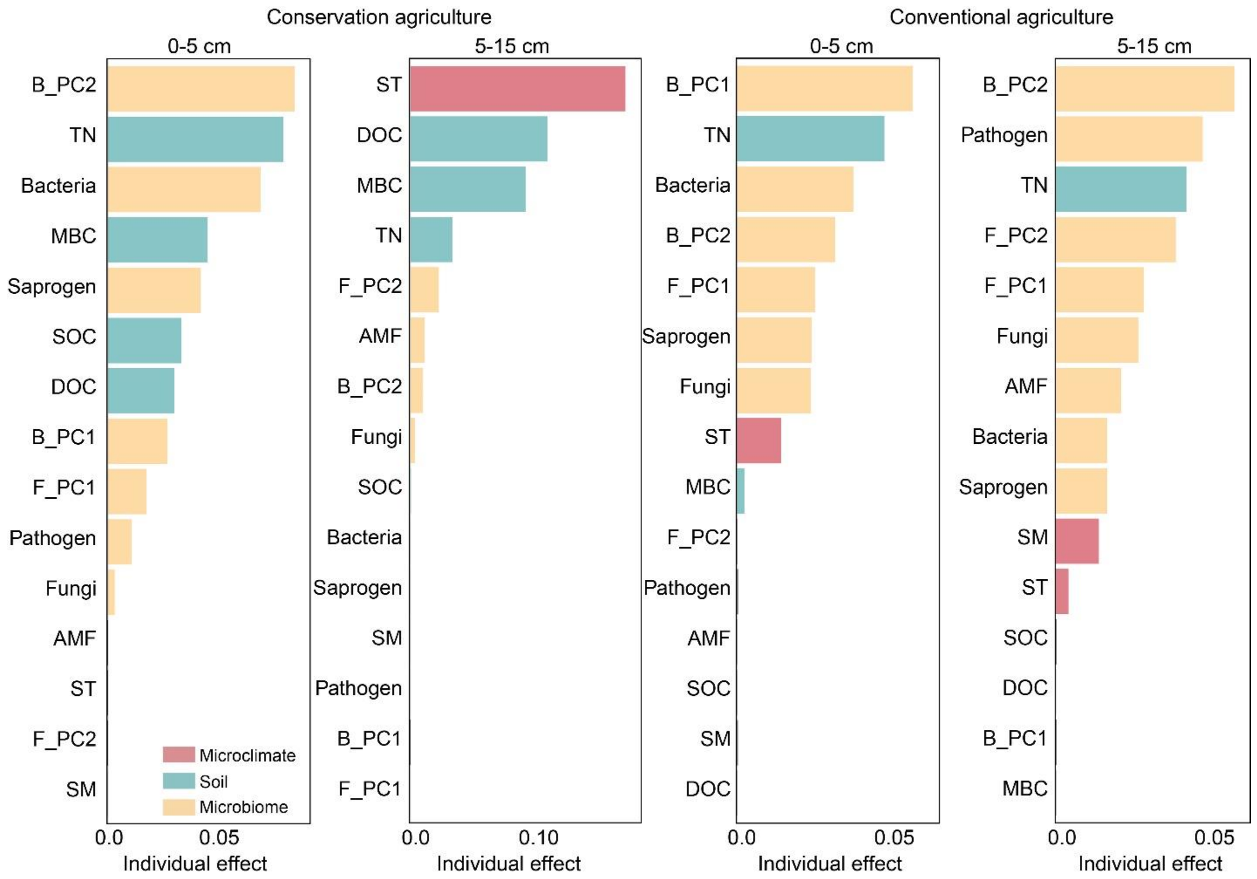

Supplementary Figure 8 Effects of abiotic and biotic factors on summer maize yield. Statistical analysis was performed using linear mixed model with sampling time as random factors. All reported *P* values result from two-sided statistical tests with **P* < 0.05, ** *P* < 0.01, and *** *P* < 0.001. ST, soil temperature; SM, soil moisture; DOC, dissolved organic carbon; MBC, microbial biomass carbon; SOC, soil organic carbon; TN, total nitrogen. Bacteria, Fungi, AMF, Saprogen, and Pathogen indicated richness of microbial group. F_PC1, F_PC2, B_PC1, and B_PC2 indicated fungal and bacterial community composition, respectively.

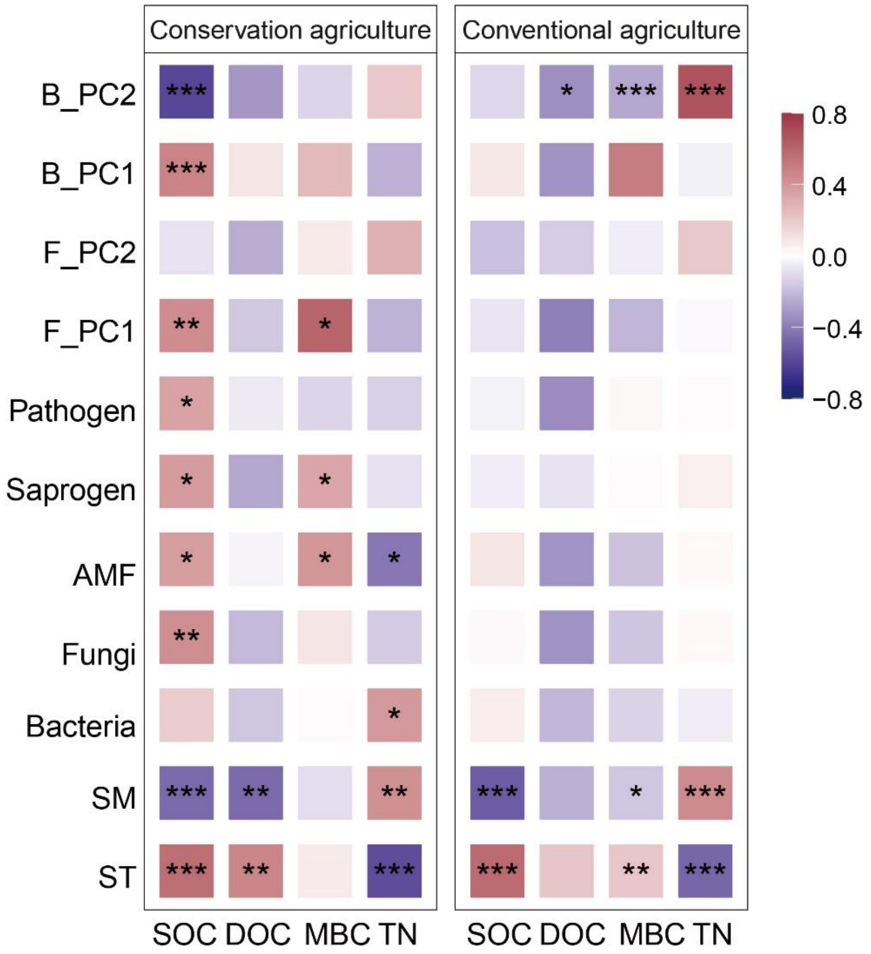

Supplementary Figure 9 Correlations between soil microclimate and microbial diversity and key indicator of soil health of 5-15 cm soil depth under conservation and conventional agriculture. The color denotes the correlation coefficient determined by the linear mixed-effects model. Statistical significance is based on Wald type II χ^2 tests. ST, soil temperature; SM, soil moisture; DOC, dissolved organic carbon; MBC, microbial biomass carbon; SOC, soil organic carbon; TN, total nitrogen. Bacteria, Fungi, AMF, Saprogen, and Pathogen indicated richness of microbial group. F_PC1, F_PC2, B_PC1, and B_PC2 indicated fungal and bacterial community composition, respectively. All reported *P* values result from two-sided statistical tests with $*P < 0.05$, $** P < 0.01$, and $*** P < 0.001$.

Supplementary Table 1 Effects of warming and management on crop yield, key soil health indicators, and soil microbial richness and by linear mixed model.

		Indicator	Management (M)			Warming(W)			M*W		
			Estimate	t value	*P* value	Estimate	t value	*P* value	Estimate	t value	*P* value
Soil conditions		Soil temperature	0.386	8.06	**0.000**	1.50	31.8	**0.000**	0.380	5.61	**0.000**
		Soil moisture	-0.735	-6.23	**0.000**	-1.61	-13.6	**0.000**	-0.520	-3.12	**0.001**
Crop yield		Winter wheat	0.151	0.868	0.092	0.468	2.69	**0.000**	0.112	0.458	0.647
		Summer maize	-0.893	-4.74	**0.000**	-0.324	-1.72	0.308	0.377	1.41	0.156
0-5 cm	Soil attributes	DOC	-1.08	-5.59	**0.000**	0.545	2.81	**0.006**	-0.346	-1.26	0.206
		MBC	-0.556	-4.20	**0.000**	0.435	3.28	0.068	-0.528	-2.82	**0.004**
		SOC	-1.80	-30.5	**0.000**	0.250	4.24	**0.005**	-0.266	-3.19	**0.001**
		TN	-1.85	-77.4	**0.000**	0.149	6.24	**0.000**	0.046	1.37	0.168
	Microbial richness	Bacteria	0.183	1.17	0.095	0.100	0.643	0.362	0.001	0.002	0.998
		Fungi	-0.067	-0.802	0.534	-0.168	-2.00	0.282	0.209	1.75	**0.049**
		AMF	-0.373	-2.81	**0.000**	-0.095	-0.717	0.364	0.020	0.107	0.915
		Saprogen	0.063	0.660	**0.010**	-0.167	-1.74	0.399	0.220	1.62	**0.050**
		Pathogen	0.034	0.396	**0.043**	-0.094	-1.10	0.925	0.178	1.46	0.143
5-15 cm	Soil attributes	DOC	-0.325	-1.25	**0.000**	1.13	4.39	**0.000**	-0.808	-2.20	**0.027**
		MBC	-0.287	-1.97	**0.035**	0.073	0.505	0.161	0.141	0.686	0.492
		SOC	-0.239	-0.969	0.787	0.770	3.11	**0.000**	0.550	1.60	0.108
		TN	0.452	1.83	-0.826	-3.35	0.229	**0.000**	-0.476	-1.39	0.164
	Microbial richness	Bacteria	0.320	1.99	**0.000**	-0.233	-1.45	0.562	0.324	1.45	0.145
		Fungi	0.029	0.285	0.742	-0.111	-1.06	**0.022**	-0.103	-0.709	0.478
		AMF	-0.125	-1.14	**0.000**	0.126	1.15	0.921	-0.257	-1.68	0.091
		Saprogen	0.520	2.56	**0.046**	-0.096	-0.474	**0.008**	-0.520	-1.85	**0.050**
		Pathogen	0.202	1.41	0.369	-0.132	-0.920	**0.013**	-0.216	-1.09	0.274

All reported *P* values result from two-sided statistical tests and significant *P* values (< 0.05) are shown in bold. DOC, dissolved organic carbon; SOC, soil organic carbon; MBC, microbial biomass carbon; TN, total nitrogen.

Supplementary Table 2 Effects of warming and management soil health indicators by two-way ANOVA.

		Management		Warming		M*W	
		F	*P*	F	*P*	F	*P*
0-5 cm	SHS	863.1	**0.000**	11.2	**0.005**	27.6	**0.000**
	MWD	120.2	**0.000**	1.64	0.223	11.5	**0.005**
	SM	66.1	**0.000**	0.365	0.556	13.8	**0.002**
	$R_{0.25}$	8.31	**0.013**	0.393	0.542	13.2	**0.003**
	BD	5.84	**0.037**	0.551	0.574	0.325	0.334
	pH	0.839	0.378	1.17	0.300	2.86	0.116
	DOC	2128.4	**0.000**	102.5	**0.000**	12.9	**0.003**
	SOC	891.1	**0.000**	1.58	0.232	3.31	0.093
	NH_4^+-N	5.53	**0.036**	2.01	0.181	0.407	0.535
	NO_3^-N	149.0	**0.000**	117.2	**0.000**	3.03	0.107
	DON	3.17	0.100	18.4	**0.001**	1.65	0.222
	TN	306.4	**0.000**	10.1	**0.007**	1.62	0.226
	AP	3.67	0.079	1113.5	**0.000**	199.6	**0.000**
	TP	113.6	**0.000**	3.12	0.102	0.023	0.883
	AK	54.5	**0.000**	6.06	**0.029**	31.8	**0.000**
	TK	0.114	0.742	0.215	0.651	0.044	0.837
	MBC	331.1	**0.000**	42.6	**0.000**	24.2	**0.000**
	MBN	1.74	0.211	40.5	**0.000**	7.15	**0.020**
5-15 cm	SHS	46.0	**0.000**	28.6	**0.000**	1.76	**0.208**
	MWD	10.3	**0.007**	6.12	**0.029**	36.4	**0.000**
	SM	92.6	**0.000**	3.28	0.095	13.4	**0.003**
	$R_{0.25}$	3.88	0.072	0.192	0.669	1.2	0.294
	BD	4.32	0.312	0.361	0.426	0.243	0.652
	pH	19.3	**0.000**	2.66	0.128	25.3	**0.000**
	DOC	1232.9	**0.000**	169.5	**0.000**	164.9	**0.000**
	SOC	3.78	0.075	16.1	**0.001**	0.139	0.715
	NH_4^+-N	559.0	**0.000**	0.599	0.454	49.6	**0.000**
	NO_3^-N	856.4	**0.000**	482.7	**0.000**	283.1	**0.000**
	DON	2.14	0.169	18.4	**0.001**	7.63	**0.017**
	TN	0.386	0.546	2.10	0.173	0.002	0.968
	AP	4.19	0.063	5.19	**0.041**	17.4	**0.001**
	TP	8.78	**0.011**	4.33	0.059	6.71	**0.023**
	AK	2.11	0.171	13.4	**0.003**	1.89	0.193
	TK	3.35	0.092	0.040	0.846	2.66	0.129
	MBC	159.4	**0.000**	92.1	**0.000**	3.97	0.069
	MBN	3.63	0.081	0.973	0.345	32.1	**0.000**

All reported *P* values result from two-sided statistical tests and significant *P* values (< 0.05) are shown in bold. SHS, soil health score; MWD, mean weight diameter; $R_{0.25}$, aggregate content with particle size larger than 0.25 mm; SM, soil moisture; BD, bulk density; DOC, dissolved organic carbon; SOC, soil organic carbon; NH_4^+-N, ammonium-nitrogen; NO_3^-N, nitrate-nitrogen; DON, dissolved organic nitrogen; TN, total nitrogen; AP, available phosphorus; TP, total phosphorus; AK, available potassium; TK, total potassium; MBC, microbial biomass carbon; MBN, microbial biomass nitrogen.

Supplementary Table 3 Effects of warming and management on soil microbial community composition by nested PERMANOVA.

	Fungi						Bacteria					
	0-5 cm			5-15 cm			0-5 cm			5-15 cm		
	F	R^2	*P*	F	R^2	*P*	F	R^2	*P*	F	R^2	*P*
Management	9.19	0.089	**0.000**	6.0	0.061	**0.000**	10.9	0.103	**0.000**	7.29	0.072	**0.000**
Warming	1.49	0.014	**0.005**	2.35	0.024	**0.000**	1.77	0.017	**0.002**	1.30	0.013	**0.002**
Warming* Management	1.07	0.010	**0.029**	0.88	0.009	0.116	0.93	0.009	**0.048**	2.34	0.023	**0.000**

All reported *P* values result from two-sided statistical tests and significant *P* values (< 0.05) are shown in bold.
