# Supplementary material for: Conservation agriculture improves soil health and sustains crop yields after long-term warming
Source: Nat Commun. 2024 Oct 10;15:8785. doi: 10.1038/s41467-024-53169-6 (PMC11467207; doi:10.1038/s41467-024-53169-6)
Supplement: Supplementary file 2 — Reporting Summary [file 41467_2024_53169_MOESM2_ESM.pdf]

nature portfolio

Corresponding author(s): Manuel Delgado-Baquerizo, Jing Tian

Last updated by author(s): Sep 20, 2024

Reporting Summary

Nature Portfolio wishes to improve the reproducibility of the work that we publish. This form provides structure for consistency and transparency in reporting. For further information on Nature Portfolio policies, see our Editorial Policies and the Editorial Policy Checklist.

Statistics

For all statistical analyses, confirm that the following items are present in the figure legend, table legend, main text, or Methods section.

n/a	Confirmed	
☐	☒	The exact sample size (*n*) for each experimental group/condition, given as a discrete number and unit of measurement
☐	☒	A statement on whether measurements were taken from distinct samples or whether the same sample was measured repeatedly
☐	☒	The statistical test(s) used AND whether they are one- or two-sided<br>*Only common tests should be described solely by name; describe more complex techniques in the Methods section.*
☐	☒	A description of all covariates tested
☐	☒	A description of any assumptions or corrections, such as tests of normality and adjustment for multiple comparisons
☐	☒	A full description of the statistical parameters including central tendency (e.g. means) or other basic estimates (e.g. regression coefficient) AND variation (e.g. standard deviation) or associated estimates of uncertainty (e.g. confidence intervals)
☐	☒	For null hypothesis testing, the test statistic (e.g. *F*, *t*, *r*) with confidence intervals, effect sizes, degrees of freedom and *P* value noted<br>*Give P values as exact values whenever suitable.*
☒	☐	For Bayesian analysis, information on the choice of priors and Markov chain Monte Carlo settings
☐	☒	For hierarchical and complex designs, identification of the appropriate level for tests and full reporting of outcomes
☐	☒	Estimates of effect sizes (e.g. Cohen's *d*, Pearson's *r*), indicating how they were calculated

Our web collection on statistics for biologists contains articles on many of the points above.

Software and code

Policy information about availability of computer code

Data collection	No software used for data collection.
Data analysis	UNOISE3, USEARCH (version 11.0.667) , and RDP Classifier were used to process the sequencing data; R (V 4.3.0) was used for statistical analyses.

For manuscripts utilizing custom algorithms or software that are central to the research but not yet described in published literature, software must be made available to editors and reviewers. We strongly encourage code deposition in a community repository (e.g. GitHub). See the Nature Portfolio guidelines for submitting code & software for further information.

Data

Policy information about availability of data

All manuscripts must include a data availability statement. This statement should provide the following information, where applicable:

- Accession codes, unique identifiers, or web links for publicly available datasets
- A description of any restrictions on data availability
- For clinical datasets or third party data, please ensure that the statement adheres to our policy

The authors declare that the data supporting the findings of this study are availability with the article and its supplementary information files. The DNA sequences of the 16S rRNA gene and ITS amplicons in this study have been deposited in the National Center for Biotechnology Information (NCBI) under project accession numbers PRJNA996529. Silva database is available at https://www.arb-silva.de/. UNITE database is available at https://unite.ut.ee/. Source data are provided in this

paper. The analysis code that supports the findings of this study is available at GitHub https://github.com/bio-carbon/warming_soil_health.

Research involving human participants, their data, or biological material

Policy information about studies with human participants or human data. See also policy information about sex, gender (identity/presentation), and sexual orientation and race, ethnicity and racism.

Reporting on sex and gender	*Use the terms sex (biological attribute) and gender (shaped by social and cultural circumstances) carefully in order to avoid confusing both terms. Indicate if findings apply to only one sex or gender; describe whether sex and gender were considered in study design; whether sex and/or gender was determined based on self-reporting or assigned and methods used. Provide in the source data disaggregated sex and gender data, where this information has been collected, and if consent has been obtained for sharing of individual-level data; provide overall numbers in this Reporting Summary. Please state if this information has not been collected. Report sex- and gender-based analyses where performed, justify reasons for lack of sex- and gender-based analysis.*
Reporting on race, ethnicity, or other socially relevant groupings	*Please specify the socially constructed or socially relevant categorization variable(s) used in your manuscript and explain why they were used. Please note that such variables should not be used as proxies for other socially constructed/relevant variables (for example, race or ethnicity should not be used as a proxy for socioeconomic status). Provide clear definitions of the relevant terms used, how they were provided (by the participants/respondents, the researchers, or third parties), and the method(s) used to classify people into the different categories (e.g. self-report, census or administrative data, social media data, etc.) Please provide details about how you controlled for confounding variables in your analyses.*
Population characteristics	*Describe the covariate-relevant population characteristics of the human research participants (e.g. age, genotypic information, past and current diagnosis and treatment categories). If you filled out the behavioural & social sciences study design questions and have nothing to add here, write "See above."*
Recruitment	*Describe how participants were recruited. Outline any potential self-selection bias or other biases that may be present and how these are likely to impact results.*
Ethics oversight	*Identify the organization(s) that approved the study protocol.*

Note that full information on the approval of the study protocol must also be provided in the manuscript.

Field-specific reporting

Please select the one below that is the best fit for your research. If you are not sure, read the appropriate sections before making your selection.

☐ Life sciences ☐ Behavioural & social sciences ☒ Ecological, evolutionary & environmental sciences

For a reference copy of the document with all sections, see nature.com/documents/nr-reporting-summary-flat.pdf

Ecological, evolutionary & environmental sciences study design

All studies must disclose on these points even when the disclosure is negative.

Study description	This study investigated the contribution of conservation agriculture compared with conventional agriculture to soil health, microbial diversity and crop yields, during eight-years' experimental warming. The study is a long-term manipulated field experiment with four treatments: conventional agriculture with and without warming (Conven-Warm and Conven-Amb), conservation agriculture with and without warming (Conserv-Warm and Conserv-Amb). Two levels of warmig (ambient and +2°C) were imposed on both conventional and conservation agriculture since 2010.
Research sample	The differences of warmed and ambient treatments under two managements represent the effects of long-term experimental warming and management interactions on soil health, microbial diversity, and crop yields.
Sampling strategy	Three composite soil samples from 0-5 and 5-15 cm soil depths were collected for each plot after harvest of winter wheat from 2010 to 2019. Composite samples were collected by hand augur consisting of five randomly chosen soil cores in each plot, then mixed together to make a composite sample. All samples were passed through a 2-mm sieve and stored at -80 °C for subsequent analysis.
Data collection	All sample collection from the experiment site was performed by authors JLT and RXH. Soil chemistry, and measurements of soil moisture, temperature, crop yield were performed by the JLT and JT following standard protocols. Soil DNA extraction and PCR were performed by JLT and JT. The amplicon sequencing and Metagenomic sequencing were performed on an Illumina Nova6000 and Illumina NovaSeq at Majorbio Bio-Pharm Technology Co., Ltd. (Shanghai, China).
Timing and spatial scale	Soil samples (0-5 cm and 5-15 cm depth) were collected after harvest of winter wheat to measure soil properties. Crop yields, soil bacterial and fungal diversity, SOC, TN, DOC, and MBC were measured annually across the eight sampling years of the experiment. Mean weight diameter (MWD), content with particle size larger than 0.25 mm (R0.25), soil moisture (SM), bulk density (BD), pH, NO3—N, NH4+-N, dissolved organic nitrogen (DON), available phosphorus (AP), total phosphorus (TP), available potassium (AK), total potassium (TK), and microbial biomass nitrogen (MBN) measured in 2020 only.

Data exclusions	No data were excluded.
Reproducibility	We provided detailed data sources, clear methods descriptions, and analysis codes to ensure the reproducibility of our results.
Randomization	Four treatments were laid out in a randomized complete block design: conventional agriculture with and without warming (Conven-Warm and Conven-Amb), conservation agriculture with and without warming (Conserv-Warm and Conserv-Amb)
Blinding	All samples taken were labeled with a single number to track samples during lab processing, but included no information as to the treatment from which it originated.

Did the study involve field work? ☒ Yes ☐ No

Field work, collection and transport

Field conditions	This study is based on a long-term climate change field trial located at North China Plain at Yucheng Comprehensive Experiment Station of Chinese Academy of Science (36° 50′ N, 116° 34′ E, elevation is 20 m). The study region has a temperate semi-arid climate with an annual mean temperature of 13.6 °C, and annual mean precipitation of 575 mm with 70% occurring between June and September. The soil type of this site is Calcaric Fluvisol (FAO-UNESCO system) with typical soil texture 12% sand, 66% silt, 22% clay, and a mean pH of 7.1. The experiment was conducted at a rotation of winter wheat (Triticum aestivum L.)-summer maize (Zea mays L.) system.
Location	The Yucheng Comprehensive Experiment Station in North China (36°51′N, 116°34′E).
Access & import/export	Project and class site use requests were completed for our study.
Disturbance	No disturbance was caused by this study.

Reporting for specific materials, systems and methods

We require information from authors about some types of materials, experimental systems and methods used in many studies. Here, indicate whether each material, system or method listed is relevant to your study. If you are not sure if a list item applies to your research, read the appropriate section before selecting a response.

Materials & experimental systems

n/a	Involved in the study
☒	☐ Antibodies
☒	☐ Eukaryotic cell lines
☒	☐ Palaeontology and archaeology
☒	☐ Animals and other organisms
☒	☐ Clinical data
☒	☐ Dual use research of concern
☒	☐ Plants

Methods

n/a	Involved in the study
☒	☐ ChIP-seq
☒	☐ Flow cytometry
☒	☐ MRI-based neuroimaging

Plants

Seed stocks	*Report on the source of all seed stocks or other plant material used. If applicable, state the seed stock centre and catalogue number. If plant specimens were collected from the field, describe the collection location, date and sampling procedures.*
Novel plant genotypes	*Describe the methods by which all novel plant genotypes were produced. This includes those generated by transgenic approaches, gene editing, chemical/radiation-based mutagenesis and hybridization. For transgenic lines, describe the transformation method, the number of independent lines analyzed and the generation upon which experiments were performed. For gene-edited lines, describe the editor used, the endogenous sequence targeted for editing, the targeting guide RNA sequence (if applicable) and how the editor was applied.*
Authentication	*Describe any authentication procedures for each seed stock used or novel genotype generated. Describe any experiments used to assess the effect of a mutation and, where applicable, how potential secondary effects (e.g. second site T-DNA insertions, mosiacism, off-target gene editing) were examined.*
